# The immunological heterogeneity of squamous cell carcinoma and adenocarcinoma of the uterine cervix: a systematic review

**DOI:** 10.1016/j.tvr.2025.200323

**Published:** 2025-07-09

**Authors:** Marije Adriana Strikwerda, Sabrina Anouck Weerstand, George Louis Burchell, Jacqueline Maria Tromp, Constantijne Helene Mom, Tanja Denise de Gruijl

**Affiliations:** a Amsterdam UMC, location Vrije Universiteit Amsterdam, Department of Gynaecological Oncology, Cancer Center Amsterdam, De Boelelaan 1117, Amsterdam, the Netherlands; b Cancer Center Amsterdam, Department of Medical Oncology, de Boelelaan 1117, Amsterdam, the Netherlands; c Amsterdam UMC, location Vrije Universiteit Amsterdam, Medical Library, De Boelelaan 1117, Amsterdam, the Netherlands

**Keywords:** Cervical cancer, Squamous cell carcinoma, Adenocarcinoma, Human papillomavirus, Tumour microenvironment, Immunotherapy

## Abstract

**Background:**

Cervical cancer is the fourth most common malignancy in women worldwide and generally driven by persistent infection with high-risk human papillomavirus. Squamous cell carcinoma (SCC) and adenocarcinoma (AC) are the two most common histological subtypes, with a relative increase in adenocarcinomas in the last decades. The immunological differences between cervical squamous cell carcinoma and adenocarcinoma remain largely unexplored. Understanding these distinctions is crucial for developing tailored therapies that can improve treatment outcomes for patients with cervical cancer. This systematic review provides an overview of the immunological features of squamous cell carcinoma and adenocarcinoma of the uterine cervix.

**Methods:**

A systematic search was performed in PubMed, Embase.com, Web of Science, and Cochrane Library. All articles addressing immunological features of squamous cell carcinoma and adenocarcinoma of the uterine cervix were reviewed and included based on predefined inclusion and exclusion criteria.

**Results:**

In total, 3207 articles were screened, of which 43 were included. Studies show that cervical squamous cell carcinomas are characterised by a more inflamed tumour microenvironment, but also contain more regulatory T cells and immune checkpoints. In contrast, adenocarcinomas are characterised by lower immune cell infiltration, contributing to its poorer prognosis and more limited response to treatment.

**Conclusion:**

The observed differences emphasize the need for further research into subtype-specific differences and distinct therapeutic strategies. For squamous cell carcinomas, future research should focus on combinatorial immune checkpoint blockade, including regulatory T cell-depleting strategies. For adenocarcinomas, oncolytic virotherapy, therapeutic vaccination, and oncogenic signalling interference should be explored.

## 1 Introduction

Cervical cancer is the fourth most diagnosed and lethal malignancy in women worldwide. In 2022, an estimated 660,000 women were diagnosed and 348,000 women died from cervical cancer [1]. Squamous cell carcinoma (SCC) and adenocarcinoma (AC) are the two most common histological subtypes of cervical cancer. SCC accounts for approximately 85 % of all cervical cancer cases and is often associated with a human papillomavirus (HPV) type-16 infection, whereas AC accounts for approximately 12 % of the cases and is often associated with an HPV type-18 infection [[2], [3], [4]]. Following HPV infection, replication of HPV in the host's cell leads to expression of the oncogenes E6 and E7. Subsequently, the E6 and E7 oncoproteins inactivate tumour suppressors p53 and retinoblastoma protein, leading to uncontrolled cell proliferation, genomic instability, and malignant progression [4,5]. Implementing HPV vaccines and population-based screening programs has led to a decrease in the cervical cancer incidence in developed countries over the last decades [4]. However, the incidence of AC has shown a relative increase compared to SCC, especially in younger women [6,7]. The 5-year overall survival rate for patients with early-stage cervical cancer is up to 95 %. However, this rate quickly drops to around 60 % for locally-advanced disease and to only 17 % for patients with metastatic disease [8,9].

Along with a shift in incidence between SCC and AC, these histologic subtypes also exhibit differences in terms of epidemiology, prognosis, mutational profiles, histologic characteristics, and their tumour immune microenvironment [9]. Whereas SCC originates from the ectocervical squamous epithelium, AC originates from the endocervical glandular epithelium [10]. In early-stage cervical cancer, survival outcomes are similar between SCC and AC following radical hysterectomy [11]. However, the increased metastatic potential of AC leads to worse survival outcomes in advanced stages, compared to SCC [[12], [13], [14]]. Nevertheless, both subtypes are treated in the same manner. Therefore, it is important to explore the root causes of these differences between histologic subtypes, in order to tailor therapeutic strategies based on tumour characteristics.

In metastasised cervical cancer, the programmed death ligand-1 (PD-L1) Combined Positive Score (CPS) is used as a biomarker for treatment selection. Patients with a CPS of 1 or higher are eligible for treatment with pembrolizumab, an antibody targeting programmed death-1 (PD-1) [15]. Studies suggest that SCC tends to express higher levels of PD-L1 compared to AC, potentially leading to more patients with SCC receiving pembrolizumab [15,16]. This may further contribute to a shift in survival rates between these histologic subtypes. Furthermore, SCC is generally considered to have a more inflamed tumour microenvironment than AC, possibly resulting in different responses to immunotherapeutic treatments [16,17]. Despite these observations, the potential immunological differences and overlaps between cervical SCC and AC remain largely unexplored. In this systematic review, we will focus on these immunological differences in the tumour immune microenvironment between SCC and AC. Understanding these distinctions is crucial for developing tailored therapies that may improve treatment outcomes for patients with cervical cancer.

## 2 Materials and methods

### 2.1 Data sources and searches

This systematic review was conducted following the Preferred Reporting Items for Systematic Reviews and Meta-Analysis (PRISMA) guidelines [18]. A systematic search was performed in the following databases: PubMed, Embase.com, Clarivate Analytics/Web of Science Core Collection and the Wiley/Cochrane Library. The timeframe within the databases was from inception to August 30th, 2024. The search included keywords and free text terms for (synonyms of) ‘Uterine Cervical Cancer’, combined with (synonyms of) ‘Adenocarcinoma’, combined with (synonyms of) ‘Squamous Cell Carcinoma’, combined with (synonyms of) ‘Immune System’. A full overview of the search terms per database can be found in Appendix A. No limitations on date or language were applied in the search. Duplicate articles were excluded using the R-package “ASYSD”, an automated deduplication tool [19] followed by manual deduplication in Endnote (X20.0.3) by the medical information specialist (G.L.B.) and in Rayyan by the investigator (M.A.S.) [20].

### 2.2 Study selection

Selection of studies was done in three steps and performed by two authors independently (M.A.S. and S.A.W.). First, records were independently screened on relevance based on title and abstract. Studies were included if a) cervical SCC and AC were investigated, and b) immunological features were described for SCC and AC, such as PD-L1 expression or immunological cell subsets (e.g. percentage of T cells). Studies were excluded if a) no comparison between SCC and AC was made; b) no outcome of interest was displayed; c) no original scientific research was conducted, including reviews, editorials, and conference abstracts; d) the articles were written in a language other than English or Dutch. Subsequently, full text articles of all selected abstracts were assessed for eligibility. Articles included through snowballing were subjected to the same screening process. Finally, discrepancies in study selection were resolved through consensus by the same two authors.

### 2.3 Study quality and risk of bias

The quality of the included studies was evaluated with an adjusted version of the OHAT risk of bias tool [21]. The studies were assessed on selection bias, confounding bias, performance bias (if applicable to the study design), attrition or exclusion bias, detection bias, and reporting bias. The studies were graded as low risk, medium risk, or high risk of bias for each type of bias. The combined risk score indicated high, medium, or low methodological quality of the included studies.

### 2.4 Data extraction

The following data was extracted from the included articles: author and year, country, study design, sample size (per histologic subtype), stage of disease, type of material, measurement technique, immune cells, markers, or cytokines, and outcome.

## 3 Results

### 3.1 Description of included studies

**Fig. 1:**
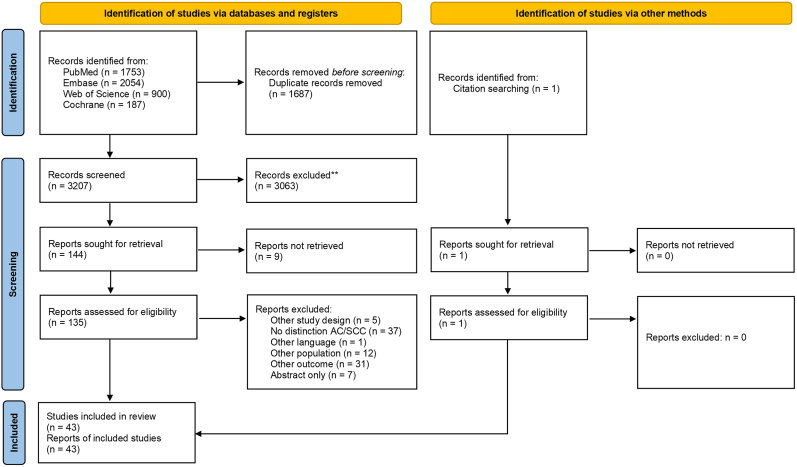
PRISMA 2020 flow diagram of the search and selection process. *Abbreviations:AC; adenocarcinoma, SCC; squamous cell carcinoma.*

The systematic literature search identified 4894 articles, of which 3207 remained after de-duplication. After screening titles and abstracts, 144 potentially eligible articles were selected for full text screening, with one additional article identified through citation searching. Eventually, 43 papers met the inclusion criteria as depicted in Fig. 1 [15,17,[22], [23], [24], [25], [26], [27], [28], [29], [30], [31], [32], [33], [34], [35], [36], [37], [38], [39], [40], [41], [42], [43], [44], [45], [46], [47], [48], [49], [50], [51], [52], [53], [54], [55], [56], [57], [58], [59], [60], [61], [62]]. More than half of the studies were performed in Asia (n = 24), followed by Europe (n = 10). The sample sizes varied from six [36] to 606 patients [62]. Per study, SCC was present in up to 491 patients [62] while AC was present in up to 146 patients [60]. Most studies included patients with early-stage or locally advanced disease (n = 19). A majority of the studies described the use of archival tumour resection material (n = 37), other studies (also) used peripheral blood (n = 7), fresh or frozen tumour samples (n = 7), or cervical cancer cell lines (n = 2). Various techniques were used for immune analyses, of which immunohistochemistry (IHC) was most commonly used (n = 35). All characteristics of the included studies are shown in Appendix B.

### 3.2 Critical appraisal

An overview of the methodological quality assessment is shown in Appendix C. The methodological quality was considered high quality (low risk of bias) in 28 studies [15,17,22,24,[26], [27], [28], [29], [30], [31], [32], [33], [34], [35], [36], [37], [38],^40^,^41^,^44^,[46], [47], [48],^51^,[58], [59], [60], [61]], moderate quality (moderate risk of bias) in 7 studies [23,25,39,42,43,56,62], and low quality (high risk of bias) in 8 studies [45,49,50,[52], [53], [54], [55],^57^]. The information from studies with a low quality were only used to reinforce data from moderate or high quality articles.

### 3.3 Immunological features of squamous cell carcinoma and adenocarcinoma of the cervix

#### 3.3.1 Infiltrating immune cell subsets

Differences in immune cell subsets within the tumour microenvironment of cervical SCC and AC were described in 15 studies. Overall, the studies agreed that cervical SCC is characterised by higher infiltration of immune cells compared to cervical AC [17,25,41,42,44,45,59]. While one study [52] reported no difference in the presence of CD45^+^ immune cells between SCC and AC, the majority of studies suggested a distinct immune landscape in SCC, with Rotman et al. [17] reporting a higher ratio of infiltrating CD45^+^ immune cells relative to EpCAM^+^ tumour cells in SCC. Specifically, tumour-infiltrating lymphocytes (TILs) were more frequent in SCC than AC, indicating a more inflamed tumour microenvironment [17,25,42,44,45,59]. Furthermore, CD3^+^ T cell infiltration was consistently higher in SCC compared to AC [17,41,42,45,52].

This higher immune cell infiltration rate in SCC was a positive predictor of disease-free survival, and hence, was associated with a favourable prognosis [45,59]. For instance, one study found that patients with SCC and a high density of tumour infiltrating immune cells exhibited the longest disease-free survival [59]. In contrast, the infiltration of immune cells in AC did not correlate with prognosis [59]. Taken together, cervical SCC appears more inflamed with higher immune cell infiltration compared to cervical AC. This difference may explain the better prognosis and higher checkpoint blockade efficacy in SCC compared to AC, as its inflamed microenvironment is likely more conducive to effective T cell mediated antitumor immunity.

While the majority of studies reported that the overall number of CD4^+^ T cells did not consistently differ between SCC and AC [17,52,56], two other studies reported a higher number of CD4^+^ T cells in SCC [32,42]. The frequency of memory CD4^+^ T cells was reported to be higher in SCC [42] as was the frequency of regulatory T cells (Tregs), suggesting a more immunosuppressive tumour microenvironment [17,32,41,42]. 10.13039/100028438Elevated forkhead box P3 (FoxP3) expression in SCC, as determined by immunohistochemistry, further supported this notion [55]. Thus, in SCC, increased infiltration by memory T cells seemed to be accompanied by increased Treg rates, possibly restraining the capacity of antitumor effector T cells.

Studies exploring differences in CD8^+^ T cell subsets between SCC and AC in cervical cancer showed conflicting results. Several studies, based on immunohistochemistry, or flow cytometry, found no significant differences in the number of CD8^+^ T cells between SCC and AC [17,29,32,41,52,56]. However, when studies based on transcriptional datasets examined specific CD8^+^ T cell subsets, SCC seemed to harbour more cytotoxic [36,52] and effector memory CD8^+^ T cells [36,42], whereas AC held a higher proportion of central memory CD8^+^ T cells [36]. Furthermore, the immune profiles of tumour-draining lymph nodes (TDLNs) analysed by flow cytometry differed between the two subtypes. In patients with cervical AC, TDLNs showed higher rates of effector and central memory CD8^+^ T cells, with fewer naïve CD8^+^ T cells. This suggests that higher densities of infiltrating memory T cells in SCC relative to AC tumours is not due to a lack of T cell priming and memory differentiation in AC TDLN [17]. Thus, while CD8^+^ T cell infiltration levels might be similar for SCC and AC, their differentiation and subset composition follow distinct patterns in both primary tumours and lymph nodes.

While natural killer (NK) cells typically do not heavily infiltrate solid tumours [63], several studies have investigated their presence in cervical SCC and AC. Findings are inconsistent: some studies that were based on transcriptional datasets reported higher [36] or similar [42,52] NK cell infiltration in SCC compared to AC, while others observed lower NK cell levels based on flow cytometry in SCC compared to AC [56]. Of note, if NK cells are not correctly identified as CD3^−^ and CD56^+^, they might be mistaken for CD3^+^CD56^+^ TILs, which are a sign of chronic stimulation and senescence, and have been observed in cervical cancer [64].

Studies on B cell infiltration in SCC and AC of cervical cancer highlight consistent differences, with SCC generally showing a higher B cell presence, based on RNA sequencing (RNAseq) [41,52], RNAseq validated by immunohistochemistry [42], or flow cytometry [56]. Indeed, SCC, rather than AC, contained a higher proportion of memory B cells and plasma cells relative to other cell types [41].

In addition, differences in myeloid cell populations between SCC and AC were observed. SCC generally showed a higher prevalence of myeloid cells based on RNAseq [41,42]. Macrophages were reported to be the dominant myeloid cell type in both SCC and AC [41], with similar frequencies observed between the two histological subtypes [41,52]. However, macrophage polarization patterns differed more distinctly. An RNAseq-based study showed that AC was associated with an abundance of tumour-associated macrophages (TAMs) expressing an M2-like signature [36]. In contrast, SCC showed upregulation of M1-like marker genes, potentially reflecting a more pro-inflammatory macrophage profile [36,42]. However, no studies could be retrieved that reported differences between SCC and AC in terms of macrophage polarization based on immunohistochemistry or flow cytometry to validate these findings. Heeren et al. [15] did find that SCC contained more PD-L1^+^ TAMs as compared with AC. These were found to express CD163, indicating an M2-like phenotype. This contradicts the findings from the RNAseq-based study.

Studies reporting dendritic cell (DC) populations in SCC and AC showed conflicting results. One study observed a higher overall presence of DCs in SCC, including more activated and plasmacytoid DCs compared to AC [42]. In contrast, other studies found no significant differences in the total number of DCs between the two histological subtypes [17,52]. However, one study reported SCC to be characterised by a higher presence of conventional type 1 DCs (cDC1), both at the transcriptional level and confirmed by flow cytometry [17]. This fits with a particular role proposed for cDC1s in T cell attraction, activation, and maintenance in the tumour microenvironment of SCC [65,66]. Indeed, rates of this cDC1 population were positively correlated with CD8^+^ T cell frequencies, supporting their role in attracting effector T cells to the tumour microenvironment. In contrast, the tumour microenvironment of AC showed a relative paucity of cDC1 and CD8^+^ effector T cells [17]. In addition, the infiltration of Langerhans cells (LCs), a type of DC predominantly found in epithelial tissues, was more frequent in SCC than in AC [45]. LCs were more commonly found in the connective tissues surrounding AC tumours, whereas in SCC, LC infiltration primarily occurred within the tumour nests, with rare presence in the surrounding connective tissue. Of note, the prognosis with LC infiltration was significantly better than without, in both SCC and AC [45]. These observations stress the importance of DCs in supporting antitumor CD8^+^ effector T cells in the tumour microenvironment, consistent with recent reports [65,67]. Thus, consistent with their higher memory T cell infiltration rates, SCC harbour higher frequencies of cDC1 and M1-like cells able to attract and support these effector T cells.

#### 3.3.2 Expression of immune checkpoint, co-inhibitory, and co-stimulatory molecules

The expression of PD-L1 in cervical tumours has been widely studied, with consistent findings indicating a higher prevalence in SCC compared to AC. Eleven studies reported significantly higher frequencies of PD-L1 expression in SCC tumours [15,22,23,36,42,43,52,55,60,62,68]. PD-L1 positivity was most often based on a CPS of 1 or higher, indicating that at least 1 % of tumour and immune cells within the tumour microenvironment express PD-L1, relative to the total number of viable tumour cells. The percentage of patients that were PD-L1 positive ranged from 33 % [31] to 87 % [43] in SCC, and from 0 % [34] to 67 % [37] in AC. In addition, twelve out of fourteen studies found higher PD-L1 expression levels in SCC than AC patients, although statistical testing was either not performed or did not reach significance [25,30,31,37,40,46,47,[49], [50], [51],^58^]. Moreover, four studies investigated PD-L1 expression with a CPS above 10, indicating that at least 10 % of cells express PD-L1 as a ratio of the total number of viable tumour cells, which was consistently seen more often in patients with SCC than AC histology [23,31,39,43]. The same trend was observed when PD-L1 expression was evaluated separately on tumour and stromal cells. In line with the previous findings, PD-L1 positivity was expressed more often on tumour [15,33,50] and stromal cells [15,33,51,61] in SCC than in AC, although in some instances differences lacked statistical significance or significance was not tested.

In addition to the presence of PD-L1, patterns of PD-L1 staining were described in two studies, though the results were ambiguous. Heeren et al. [15] reported that diffuse PD-L1 staining was observed more often in SCC, whereas marginal PD-L1 staining occurred more frequently in AC. However, Omenai et al. [47] reported AC tumours to exclusively exhibit diffuse PD-L1 staining patterns. In SCC, the presence of PD-L1^+^ cordons surrounding tumour fields was noted; which have been described as aggregates of PD-L1^+^ macrophage-like cells at the tumour-stroma interface [15]. These PD-L1^+^ immune cells might have an immunosuppressive effect by inhibiting T cell function and/or migration.

Relating PD-L1 expression patterns to survival outcomes, SCC tumours with negative or diffuse PD-L1 expression tended to have a worse disease-free and disease-specific survival, compared to those with marginal PD-L1 expression; the latter possibly signalling local interferon-gamma (IFN-γ) release by effector T cells [15]. Interestingly, in patients with AC, PD-L1 positive tumours showed a worse overall survival compared to patients with PD-L1 negative tumours [33]. This was confirmed by the finding that the presence of PD-L1^+^ TAMs was associated with worse disease-specific survival in AC, but not in SCC [15].

In addition to PD-L1, PD-L2 expression was also found to be higher in SCC than in AC tumours [42,51]. As for the PD-L1/L2 ligand PD-1, studies consistently reported higher expression in SCC compared to AC tumours [17,42]. Specifically, patients with SCC exhibited higher expression levels of PD-1 on conventional CD4^+^ T helper cells and CD8^+^ T cells in both primary tumours and draining lymph nodes, indicating activated but possibly also more exhausted T cells [17].

In addition to the PD-L1/L2-PD-1 axis, several other immune checkpoint and costimulatory molecules were found to differ between cervical SCC and AC. Notably, gene expression analysis showed that SCC tumours exhibited higher expression of Cytotoxic T-Lymphocyte Associated protein 4 (CTLA-4) [36,52]. Several other studies confirmed this finding by immunohistochemistry and multicolor immunofluorescence [33,42]. In addition, in TDLN of patients with SCC, higher expression levels of CTLA-4 were found on conventional CD4^+^ T-helper cells than in patients with AC [17].

The immune checkpoint lymphocyte-activation gene 3 (LAG-3) was reported to be expressed at higher levels on CD4^+^ and CD8^+^ T cells in TDLN of SCC patients compared to AC, but no significant differences were found in primary tumours [17,42]. In contrast, T cell immunoglobulin and mucin-domain containing-3 (TIM-3) expression was higher on CD4^+^ and CD8^+^ T cells in both primary tumours and draining lymph nodes of SCC patients compared to AC patients. In addition, SCC tumours exhibited a higher frequency of CD4^+^ and CD8^+^ T cells co-expressing TIM-3 with PD-1, and also LAG-3 co-expressed more often with other checkpoint molecules on CD8^+^ T cells, suggesting a more exhausted immune state in SCC than in AC [17]. The expression of T cell immunoreceptor with immunoglobulin and ITIM domain (TIGIT) in SCC and AC was inconsistent, with one study reporting higher expression in SCC [42], whereas another found higher expression of TIGIT in AC tumours [48].

The expression of other B7 family molecules also varied between SCC and AC. B7-H3 was reported to be more frequently expressed on tumour and stromal cells in SCC than in AC [60,62], although one study found no difference between the two histologic subtypes [39]. In addition, B7-H4 was expressed more frequently in SCC than in AC [62]. Regarding the immune checkpoint V-domain Ig suppressor of T cell activation (VISTA), one study found no difference between SCC and AC [38], whereas another reported higher expression of VISTA on both immune and tumour cells in SCC [62].

With regard to the costimulatory molecule CD80, macrophage-like populations expressing PD-L1 and PD-L2 in SCC showed higher levels of CD80 co-expression compared to AC. In AC, more infiltrating macrophages expressed PD-L1 and PD-L2, but with lower or absent CD80 expression, indicating a more immunosuppressive microenvironment [51]. It has been suggested that simultaneous expression of PD-L1 and CD80 can inactivate PD-L1 while preserving the co-stimulatory function of CD80 [69]. Consequently, while PD-L1-expressing macrophages were more common in SCC, these macrophages in AC may be more effective at suppressing T cells due to the absence of CD80. This suggests that macrophages in AC may be more effective in predicting the efficacy of PD-(L)1 blockade and may explain the association of TAMs with shorter survival in AC [51]. Finally, immune-related relative gene expression revealed that SCC was associated with a higher enrichment of costimulatory molecules ICOS and CD28 when compared to AC [36].

Overall, these studies clearly indicate a higher state of immune activation in SCC, potentially regulated by elevated levels of immune checkpoint expression. This suggests that immune checkpoint blockade may offer greater therapeutic benefit in SCC compared to AC.

#### 3.3.3 Cytokines and chemokines

Differences in cytokine and chemokine profiles may indicate differences in the immune activation and infiltration status of SCC versus AC. CXC chemokine receptor (CXCR) type 4 (CXCR4) expression was significantly higher in SCC compared to AC, and was associated with lymph node metastasis [24]. Similarly, the expression of CC chemokine receptor (CCR) type 7 (CCR7), the receptor for the chemokine (CC motif) ligands (CCL) type 19 (CCL19) and 21 (CCL21), considered to mediate naïve lymphocyte trafficking, was significantly elevated in SCC compared to AC [24]. In addition, the chemokine (CXC motif) ligands (CXCL) 9 (CXCL9), 10 (CXCL10), and 11 (CXCL11), known to attract effector immune cells, were expressed at higher levels in SCC, correlating with higher effector CD8^+^ T cell scores and cDC1 transcriptional signatures, indicative of enhanced immune cell recruitment [17,36]. In contrast, AC exhibited significantly lower levels of these chemokines, consistent with reduced T cell trafficking and a less T cell inflamed tumour microenvironment [17]. Moreover, Li C et al. [36] found that TAMs from AC showed an upregulation of CCL18, CCL13, CXCL3, and CXCL5 expression, which have been described to attract, among others, cancer-associated fibroblasts (CAFs), neutrophils, and Tregs, and have been related to metastasis and poorer survival outcomes in several cancer types [[70], [71], [72], [73], [74], [75], [76], [77], [78], [79]]. On the contrary, macrophages in SCC showed higher expression of CXCL9 and CXCL10, again consistent with the attraction of effector T cells. In addition, CCL4, a chemokine involved in attracting cDC1, was markedly elevated in SCC. In contrast, AC showed increased activation of the Wnt/β-catenin pathway, which may impede CCL4-mediated cDC1 recruitment, and down-stream CXCL9/10/11-mediated effector T cell recruitment, thus possibly dampening antitumor immune activity [17].

**Fig. 2:**
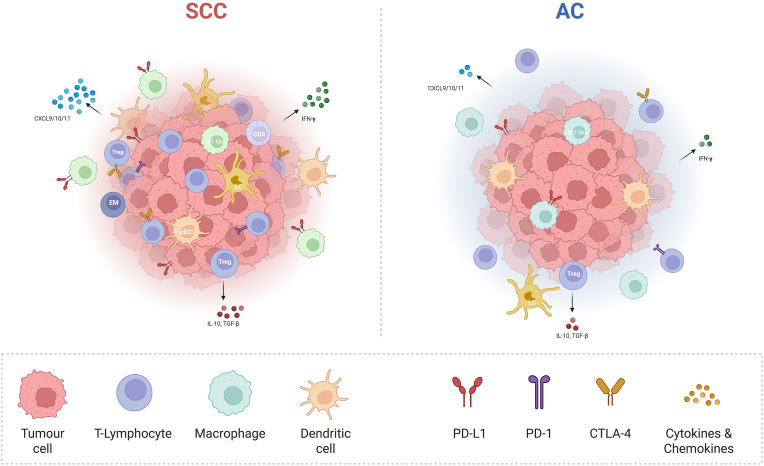
Schematic overview of the different immune characteristics of SCC and AC of the cervix. SCC shows enhanced immune cell infiltration, with more TILs and pro-inflammatory (M1-like) macrophages, whereas AC shows more macrophages polarised towards an immunosuppressive (M2-like) phenotype. SCC is characterised by more immunosuppressive Tregs, possibly restraining effector T cells. SCC tumours exhibit increased expression of immune checkpoints (indicated in the figure as PD-L1, PD-1, and CTLA-4), suggesting tumour immune evasion and T cell exhaustion. cDC1 are more abundant in SCC, which attract effector T cells and are associated with response to immune checkpoint blockade. LCs, indicative of improved prognosis, infiltrate tumour nests in SCC but reside in the surrounding stroma of AC. The levels of CXCL 9, CXCL10, and CXCL11, are higher in SCC, indicative of enhanced T cell recruitment as compared to AC where it is consistent with a less T cell inflamed tumour microenvironment. The expression of IFN-γ, associated with antitumor immunity, was found to be higher in SCC compared to AC, as were the levels of IL-10 and TGFβ. The inflamed microenvironment in SCC likely supports effective T cell mediated antitumor immunity and may explain higher checkpoint blockade efficacy compared to AC. The expression of multiple immune checkpoints and high Treg rates in SCC support the validity of exploring checkpoint blockade combinations and Treg-depleting strategies, such as blockade of PD-1 and CTLA-4. The immune excluded and immune deserted immunotype in AC may require immunotherapeutic strategies to prime and recruit antitumor T cells, such as therapeutic vaccines, oncolytic viruses or modulation of oncogenic signalling pathways. *Abbreviations: AC; adenocarcinoma. cDC1; conventional type 1 dendritic cells. CXCL; chemokine (C-X-C motif) ligands. Cytotoxic T-Lymphocyte Associated protein-4. EM; effector memory. IFN-γ; interferon-gamma. IL-10; interleukin-10. LC; Langerhans cells. PD-1; programmed death-1. PD-L1; programmed death ligand-1. SCC; squamous cell carcinoma. TGFβ; transforming growth factor beta. TILs; tumour-infiltrating lymphocytes. Tregs; regulatory T cells*.

Interleukin-I receptor antagonist (IL-1ra), a naturally occurring antagonist for interleukin (IL) 1 with immunosuppressive effects, was significantly elevated in SCC compared to AC. These elevated levels in SCC were correlated with lymph node metastasis and poorer prognosis in SCC patients. In AC, however, IL-1ra levels had no impact on disease-free survival [28]. Moreover, the secretion of IL-6, a pro-inflammatory cytokine, but with possible immune suppressive effects on myeloid cells [80], was higher in SCC than in AC serum and cell lines [57]. Similarly, immune suppressive IL-10 expression levels were significantly higher in SCC [42]. The expression of IFN-γ, associated with antitumor immunity, was found to be higher in SCC compared to AC [36]. However, the correlation between the IFN-γ signalling pathway and PD-L1/PD-L2 expression suggested a stronger survival benefit for SCC patients with high levels of IFN-γ and PD-L1/PD-L2, compared to AC, where the survival advantage was less pronounced [51]. Finally, the majority of studies agreed that the expression levels of TGFβ were significantly upregulated in SCC compared to AC [36,42]. Overall, the higher cytokine and chemokine profiles in SCC, as compared to AC, further support the earlier observation of increased inflammation and immune cell infiltration in SCC. A schematic overview of the different immune characteristics of SCC and AC, including infiltrating immune cells, expression of immune checkpoints, and presence of cytokines, is shown in Fig. 2.

#### 3.3.4 HLA expression patterns

Tumours can escape destruction by cytotoxic cells through downregulation of classical class-I human leukocyte antigens (HLA) (HLA-A, -B, and -C). These classical HLA molecules are responsible for tumour-antigen presentation mediating recognition by cytotoxic T cells. Indeed, it has been shown that HLA class-I downregulation is correlated to a decrease in the number of CD8^+^ TILs [81]. Classical HLA expression patterns differed between cervical SCC and AC, particularly in metastatic lymph nodes. Primary tumours and metastatic lymph nodes of SCC demonstrated more complete loss of HLA-A as compared to AC. In AC however, there were more lymph nodes with complete loss of HLA-B and -C, which was correlated with parametrial invasion [27]. These differences in HLA expression patterns may contribute to distinct immune evasion mechanisms between SCC and AC.

In addition, tumour cells can increase the expression of non-classical HLA class I molecules, such as HLA-E and HLA-G. These can interact with inhibitory receptors on cytotoxic T cells, NK cells, and myeloid cells, leading to decreased activity with potential tumour progression as a result [42,53]. AC demonstrated a greater HLA-E-mediated immune inhibitory response of CD4^+^ and CD8^+^ T cells, potentially contributing to T cell exhaustion [48]. Moreover, in SCC, loss of HLA-A or complete loss of total classical class I HLA, combined with HLA-G expression was associated with poorer disease-specific and disease-free survival [27]. This is consistent with significant immune editing of SCC tumours and indicates evolutionary pressure provided by anti-tumour effector T cell activity.

Finally, cervical tumours tend to up-regulate class II HLA molecules (HLA-DR, -DP, and -DQ) that can present peptides to CD4^+^ T helper cells to initiate cell-mediated immune responses [42,53]. Interestingly, HLA-DRα expression patterns exhibited notable differences between SCC and AC. Samuels et al. [53] reported significantly higher expression in AC compared to SCC. Moreover, high HLA-DRα expression in AC was associated with improved disease-free and disease-specific survival, while no such survival benefit was observed in SCC [53]. Possible mechanisms underlying this observation as yet remain obscure.

## 4 Discussion

This systematic review provides a comprehensive overview of the immunological differences between the two major histological subtypes of cervical cancer: squamous cell carcinoma and adenocarcinoma. While these subtypes differ in terms of anatomical cancer origin (i.e. endocervix versus ectocervix), types of HPV infection, therapeutic responses, prognosis, and survival, patients with AC are still treated similarly to those with SCC. However, evidence suggests that the immune escape mechanisms vary between the two subtypes, which could inform the development of tailored subtype-specific immunotherapeutic strategies. Given the viral aetiology of cervical cancer, immunotherapy offers a highly promising treatment approach. Currently, PD-1 blockade is the only FDA-approved immunotherapeutic strategy for cervical cancer, but it is restricted to patients with PD-L1 positive tumours. Given these insights, we explored how the distinct immunological profiles of SCC and AC could guide the future design of tailored immunotherapeutic approaches.

We found that the tumour microenvironment in SCC is characterised by higher levels of immune cell infiltration compared to AC, with notably greater numbers of T cells and macrophages, particularly those with a pro-inflammatory phenotype. These findings suggest a more inflamed tumour microenvironment in SCC compared to AC. However, despite the increased immune cell infiltration, SCC tumours also exhibit increased expression of immune checkpoints, possibly indicating tumour immune evasion and increased T cell exhaustion in an effort to evade the apparent inflammatory T cell response. In contrast, AC exhibits a less immune-infiltrated tumour microenvironment, with lower levels of lymphocytes and macrophages that seem to naturally polarize more towards an immunosuppressive phenotype.

One possible explanation for the increased T cell infiltration in SCC might be a higher number of T-cell-attracting chemokines in this subtype. In AC, lower levels of these chemokines were found, which may explain the decreased T cell presence in the tumour microenvironment. This reduced chemokine signalling in AC may be attributed to impaired cDC1 recruitment, which is one of the crucial producers of these T cell attracting chemokines. Moreover, the presence of cDC1 has been associated with response to immune checkpoint blockade and improved survival across different tumour types [82,83]. Impaired recruitment of cDC1 might be due to activation of the Wnt/β-catenin pathway [66]. Enhancing cDC1 function and promoting their recruitment to the tumour could improve immune cell infiltration in AC and induce a more inflamed phenotype, potentially making it more responsive to immune checkpoint blockade and other forms of immunotherapy. This could possibly be achieved by administration of Wnt-inhibitors or (oncolytic) viruses that encode constitutively active GSK3β, targeting the Wnt/β-catenin pathway [17].

Another explanation for the reduced T cell infiltration in AC might be its lower intrinsic immunogenicity compared to SCC, possibly due to the difference in HPV subtype infection. Since HPV type-16 is usually associated with SCC and HPV type-18 accounts for most AC cases [84], the observed differences in this review might reflect the impact of HPV subtypes rather than histological subtypes. Although both HPV ype-16 and HPV type-18 interfere with DC activation and migration, and with NF-kβ- and IRF3-mediated signalling, thus hampering activation of both innate and adaptive immunity [85], very few head-to-head comparative analyses have been performed to unveil differences in the immune modulatory effects of these different HPV genotypes. Nevertheless, a few examples of such differential effects have been reported. For instance, high-risk HPV infection can increase stemness in malignant cells, with highest stemness scores in (HPV18-associated) AC compared to SCC. This might result in a more inherently inhibitory tumour microenvironment characterised by exhausted and less cytotoxic CD8^+^ T cells [41]. Moreover, HPV type-16 infection, more commonly associated with SCC, has been described to stimulate more clonal T cell expansion with more overlapping clonotypes between CD4^+^ and CD8^+^ T cell subsets, indicating a more coordinated, potentially impactful, immune response compared to HPV type-18, which is typically associated with AC [48]. Innate immunity against HPV types is likely initiated by specialised double-stranded viral DNA sensors like Toll-like receptor 9 (TLR9). Indeed, CpG motifs in the HPV genome were shown to bind TLR9 and the HPV16 E6 and E7 oncoproteins were shown to interfere with TLR9 expression in infected keratinocytes, thus likely sabotaging an innate antiviral response. In contrast, HPV18 E6 and E7 were less efficient in effecting this transcriptional inhibition of TLR9 [86]. In aggregate, these reports suggest either a stronger HPV16/SCC-associated T cell response calling for more effective immune escape or a more inherently immune suppressed tumour microenvironment associated with HPV18/AC.

While SCC may show a more inflamed phenotype compared to AC, this subtype also contains a higher number of Tregs compared to AC, which are known for their immunosuppressive functions. The presence of Tregs in SCC has been linked to poor survival outcomes [32]. In contrast, in AC, Tregs are associated with improved survival, likely due to the simultaneous infiltration of effector T cells, an overriding prognostic factor in this less inflamed histological subtype [87]. Immune checkpoint blockade with anti-CTLA-4, such as ipilimumab, could benefit both SCC and AC. Anti-CTLA-4 antibodies have shown to reduce intratumoral Treg rates [[88], [89], [90]], which may promote antitumor activity in SCC. In AC, the abundant CD8^+^ T cells present in TDLN could also be targeted by anti-CTLA-4 as this prevents binding of CTLA-4 to its ligands CD80/86 thereby allowing more effective co-stimulation and activation of T cells in the lymph nodes. From this perspective, local immunotherapy could be an interesting approach to more directly leverage these T cells in the TDLN, thereby inducing immune activation and recruitment towards the tumour [17].

In addition to the more inflamed phenotype of SCC compared to AC, there is consensus that SCC expresses more and higher levels of PD-L1. This apparent upregulation of PD-L1 may be attributed to the greater number of infiltrating T cells in SCC, which produce IFN-γ and thereby induce PD-L1 expression [17]. This can be confirmed by the higher levels of IFN-γ reported in SCC relative to AC [36]. Higher levels of PD-L1 might suggest that SCC tumours are more prone to immune evasion but may also make them a more suitable target for immune checkpoint blockade with anti-PD-(L)1. It is worth noting that immune checkpoint blockade has been shown to yield responses even in patients with PD-L1-negative tumours, highlighting the complex role of PD-L1 as a predictive biomarker for immune checkpoint blockade [51,[91], [92], [93]].

In addition to PD-L1, SCC displays elevated levels of other immune checkpoints, such as PD-1, CTLA-4, and TIM-3. The upregulation of PD-1 indicates T cell activation in the tumour microenvironment, while co-expression of CTLA-4 and TIM-3 may suggest T cell exhaustion. PD-1 blockade can reinvigorate (progenitor-)exhausted T cells and has proven efficacious in patients with cervical cancer. The Keynote-826 study, that combined pembrolizumab with chemotherapy and bevacizumab, has led to FDA-approval of pembrolizumab as first-line treatment for advanced cervical cancer patients (with a PD-L1 CPS of one or higher) [94]. However, the objective response rate to anti-PD-1 monotherapy in previously treated recurrent ormetastasised cervical cancer patients remains relatively low at a maximum of 26 % [46,92,95]. The co-expression of other immune checkpoints in SCC may indicate a greater potential for response to combinatorial immune checkpoint blockade, such as anti-PD-1 with anti-CTLA-4. In cervical cancer patients, this combination has led to objective response rates of up to 40 % in clinical trials [[96], [97], [98]]. However, many patients experienced immune-related adverse events, demonstrating the importance to stratify patients based on their possible benefit from this dual immune checkpoint blockade. However, in many clinical trials, the investigation of biomarkers is mainly generalised and not focused on specific histologic subtypes [41]. Moreover, trials investigating immune checkpoint blockade do not focus on histologic subtypes or include solely patients with SCC [92,95,96]. As a result, the observed differences in immunotherapy efficacy between SCC and AC are still not well understood. Nevertheless, given the rising incidence of AC relative to SCC, its lower immunogenicity and poorer prognosis, it is crucial to explore specific therapeutic strategies and companion predictive biomarkers for this cervical cancer subtype. Understanding the tumour immune microenvironment of cervical cancer subtypes, together with their genetic landscapes (and how the latter may inform the former), could guide further research to identify potentially beneficial combination treatments for both SCC and AC of the cervix.

Interestingly, the distinct immunological profiles observed for SCC and AC in cervical cancer align with findings reported in other malignancies with these histological subtypes, such as lung cancer, oesophageal cancer, and head-and-neck cancer [[99], [100], [101]]. For instance, in SCC of the lung, a higher immune cell infiltration, including CD8^+^ T cells and macrophages, was observed compared to lung AC, which often exhibited immune desert features [99]. Similar to cervical cancer, lung and head-and-neck SCC is associated with elevated PD-L1 expression and a more inflamed tumour microenvironment compared to AC, rendering it more responsive to PD-(L)1 blockade. In contrast to our findings in cervical cancer, AC of the lung often shows higher expression of immune checkpoints such as TIGIT and VISTA, contributing to its immunosuppressive phenotype [99,100]. In oesophageal cancer, SCC also exhibits higher levels of immune cell infiltration and checkpoint expression compared to AC [100]. This is in keeping with the more favourable outcomes observed in SCC patients undergoing immunotherapy. Finally, the immune-excluded phenotype observed in oesophageal AC parallels findings in cervical AC, suggesting shared immunosuppressive mechanisms such as poor chemokine signalling and Wnt pathway activation [100].

While this review offers valuable insights into the immune microenvironment of cervical SCC and AC, several limitations must be acknowledged. The included studies exhibit methodological variability, including differences in sample types (e.g. tumour versus TDLN, and paraffin embedded versus fresh tumour material, see Table B.1), immune analysis techniques, and patient populations, which complicates direct comparisons and may introduce bias. A limitation in assessing PD-L1 is the use of different scoring methods or cut-off values across studies, including studies that use other methods than the CPS. In addition, PD-L1 scoring is subject to considerable inter-observer variability [102]. Moreover, due to the spatial heterogeneity of PD-L1 expression within tumours, sampling bias might have impacted the results [103,104]; by the same token heterogeneous immune infiltration patterns may have led to sampling bias. Another limitation is that most studies focused on immune cell abundance and marker expression without evaluating functional aspects, limiting our understanding of the underlying biology defining the differences between SCC and AC. Moreover, we did not separate results based on disease stage, despite advanced-stage patients typically harbouring more immunosuppressed tumours and displaying higher levels of systemic immune suppression compared to those with early-stage cervical cancer. Since most resected materials came from early-stage patients, as reflected in the studies included, this may have limited the generalizability of our findings to all disease stages. Unfortunately, aside from tumour-infiltrating lymphocytes and PD-L1 expression, only a limited number of studies reported on identical immunological features, making it difficult to draw strong conclusions.

This review also has several notable strengths. First, we included a wide range of immunological characteristics, by which we tried to provide a detailed understanding of the tumour immune microenvironment in cervical SCC and AC. By specifically comparing these histologic subtypes, this study addresses a critical gap in cervical cancer research, providing insights into the distinct immunological profiles and shedding light on the factors contributing to the poorer treatment response of AC compared to SCC. Future research should focus on combining the immunologic and genetic landscapes of the different histologic subtypes of cervical cancer. This approach is essential to unravel the intricate complexity and diversity of the tumour microenvironments of both histologic subtypes. Ultimately, this knowledge should guide the development and testing of different therapeutic strategies tailored to histologic subtype in order to improve the prognosis of cervical cancer patients.

## 5 Conclusions

This systematic review highlights the distinct immunological profiles of SCC and AC of the cervix. SCC is characterised by a more inflamed tumour microenvironment, which may enhance its responsiveness to immune checkpoint blockade. In contrast, AC exhibits lower immune cell infiltration and immunogenicity, contributing to its poorer prognosis and a more limited response to immune checkpoint blockade. These differences emphasize the need for subtype-specific therapeutic strategies. The expression of multiple immune checkpoints and high Treg rates in SCC, support the validity of exploring immune checkpoint inhibitor combinations and Treg-depleting strategies for this subtype. While immune checkpoint blockade shows promise for SCC, the limited response in AC and the underrepresentation of AC in clinical trials, highlight the need for further research to optimize therapy for both subtypes. The more immune excluded and immune deserted immunotype observed in the AC tumour microenvironment points to the need for immunotherapeutic strategies aiming at *de novo* priming and attraction of antitumor T cells, like therapeutic vaccination, oncolytic virotherapy or oncogenic signalling interference to modulate the immune infiltrate. A better understanding of the immunological landscape of both SCC and AC will be crucial for developing more effective targeted treatments and improving patient outcomes.

## CRediT authorship contribution statement

**Marije Adriana Strikwerda:** Writing – review & editing, Writing – original draft, Visualization, Methodology, Investigation, Data curation, Conceptualization. **Sabrina Anouck Weerstand:** Writing – original draft, Investigation, Data curation. **George Louis Burchell:** Writing – review & editing, Methodology. **Jacqueline Maria Tromp:** Writing – review & editing. **Constantijne Helene Mom:** Writing – review & editing, Supervision. **Tanja Denise de Gruijl:** Writing – review & editing, Supervision, Conceptualization.

## Declaration of competing interest

The authors declare that they have no known competing financial interests or personal relationships that could have appeared to influence the work reported in this paper.

## Figures and Tables

**Table A.1:** Search Strategy PubMed.

Search	Query	Results
#5	Search: #1 AND #2 AND #3 AND #4	1,753
#4	Search: "Immune System"[Mesh] OR "Immune Checkpoint Proteins"[Mesh] OR "Immune Checkpoint Inhibitors"[Mesh] OR "Cytokines"[Mesh] OR "Cancer-Associated Fibroblasts"[Mesh] OR "Lymphocyte Count"[Mesh] OR "Leukocytes"[Mesh] OR "Immunohistochemistry"[Mesh] OR "Dendritic Cells/immunology"[Mesh] OR "CD8-Positive T-Lymphocytes/immunology"[MeSH] OR "programmed cell death 1 receptor"[Mesh] OR "Flow Cytometry"[Mesh] OR "Single-Cell Analysis"[Mesh] OR "Inflammation"[Mesh] OR "Neutrophils"[Mesh] OR "Killer Cells, Natural"[Mesh] OR "Natural Killer T-Cells"[Mesh] OR "Plasma Cells"[Mesh] OR "Monocytes"[Mesh] OR "Antigen-Presenting Cells"[Mesh] OR "Tumour Microenvironment/genetics"[Mesh] OR "Tumour Microenvironment/immunology"[Mesh] OR "CD4-CD8 Ratio"[Mesh] OR "Tumour-Associated Macrophages"[Mesh] OR "Sequence Analysis, RNA"[Mesh] OR "Sequence Analysis, DNA"[Mesh] OR "Biomarkers, Tumour"[Mesh] OR "Biomarkers"[Mesh] OR Immun∗[tiab] OR "programmed death ligand∗"[tiab] OR "programmed cell death ligand"[tiab] OR "programmed death 1∗"[tiab] OR "programmed cell death 1∗"[tiab] OR Lymphoid∗[tiab] OR TLS[tiab] OR Basophil∗[tiab] OR Neutrophil∗[tiab] OR "Interferon gamma"[tiab] OR Cytokine∗[tiab] OR Chemokine∗[tiab] OR CAF[tiab] OR Eosinophil∗[tiab] OR "Toll-like receptor∗"[tiab] OR IHC[tiab] OR Immunohistochem∗[tiab] OR FFPE[tiab] OR "Formalin-fixed paraffin embedded"[tiab] OR TIL[tiab] OR lymphocyt∗[tiab] OR "tumour infiltrat∗"[tiab] OR "tumour infiltrat∗"[tiab] OR "PD 1"[tiab] OR "PD L1"[tiab] OR PD1[tiab] OR PDL1[tiab] OR "T cell∗"[tiab] OR "CTLA∗"[tiab] OR CTLA4∗[tiab] OR "dendritic cell∗"[tiab] OR CD4[tiab] OR CD8[tiab] OR "CD 4∗"[tiab] OR "CD 8∗"[tiab] OR "natural killer cell∗"[tiab] OR "NK cell∗"[tiab] OR "T helper cell∗"[tiab] OR "th cell∗"[tiab] OR "T cell∗"[tiab] OR "Treg∗"[tiab] OR CTL[tiab] OR macrophage∗[tiab] OR leukocyt∗[tiab] OR "B cell∗"[tiab] OR "myeloid∗"[tiab] OR inflammat∗[tiab] OR Immuno∗[tiab] OR "Flow cyto∗"[tiab] OR "Plasma cell∗"[tiab] OR "Monocyte∗"[tiab] OR Fibroblast[tiab] OR Interleukin∗[tiab] OR "Antigen presenting"[tiab] OR	6,401,331
#3	Search: (("Carcinoma, Squamous Cell"[Mesh] OR "squamous cell carcinoma∗"[tiab] OR "squamous carcinoma∗"[tiab] OR "Epidermoid Carcinoma∗"[tiab] OR "Planocellular Carcinoma∗"[tiab] OR "Plano cellular Carcinoma∗"[tiab] OR Squamous∗[tiab]) AND (cervical∗[tiab] OR cervix∗[tiab] OR Endocervical[tiab]))	29,578
#2	Search: (("Adenocarcinoma"[Mesh] OR "adenocarcinoma"[tiab]) AND (cervical∗[tiab] OR cervix∗[tiab] OR Endocervical[tiab]))	15,324
#1	Search: "Uterine Cervical Neoplasms"[Mesh] OR "cervical cancer∗"[tiab] OR "cervical tumo∗"[tiab] OR "cervical malignanc∗"[tiab] OR "cervical neoplasm∗"[tiab] OR "cervical carcinoma∗"[tiab] OR "cervical adenocarcinoma"[tiab] OR "cervical intraepithelial neoplasia"[tiab] OR "cervical metasta∗"[tiab] OR "cervix cancer∗"[tiab] OR "cervix tumo∗"[tiab] OR "cervix malignanc∗"[tiab] OR "cervix neoplasm∗"[tiab] OR "cervix carcinoma∗"[tiab] OR "cervix adenocarcinoma"[tiab] OR "cervix squamous cell carcinoma∗"[tiab] OR "cervix intraepithelial neoplasia"[tiab] OR "cervical squamous cell carcinoma∗"[tiab]	118,506

**Table A.2:** Search Strategy Embase.com.

Search	Query	Results
#6	#5 AND ('article'/it OR 'article in press'/it OR 'review'/it)	2054
#5	Search: #1 AND #2 AND #3 AND #4	2664
#4	'immune checkpoint protein'/exp OR 'immune checkpoint inhibitor'/exp OR 'cytokine'/exp OR 'cancer associated fibroblast'/exp OR 'leukocyte count'/exp OR 'leukocyte'/exp OR 'immunohistochemistry'/exp OR 'biologic factors and agents acting on the immune system'/exp OR 'flow cytometry'/exp OR 'single cell analysis'/exp OR 'inflammation'/exp OR 'RNA sequencing'/exp OR 'DNA sequencing'/exp OR 'tumour marker'/exp OR 'biological marker'/exp OR (Immun∗ OR "programmed death ligand∗" OR "programmed cell death ligand" OR "programmed death 1∗" OR "programmed cell death 1∗" OR Lymphoid∗ OR TLS OR Basophil∗ OR Neutrophil∗ OR "Interferon gamma" OR Cytokine∗ OR Chemokine∗ OR CAF OR Eosinophil∗ OR "Toll-like receptor∗" OR IHC OR Immunohistochem∗ OR FFPE OR "Formalin-fixed paraffin embedded" OR TIL OR lymphocyt∗ OR "tumour infiltrat∗" OR "tumour infiltrat∗" OR "PD 1″ OR "PD L1" OR PD1 OR PDL1 OR "T cell∗" OR "CTLA∗" OR CTLA4∗ OR "dendritic cell∗" OR CD4 OR CD8 OR "CD 4∗" OR "CD 8∗" OR "natural killer cell∗" OR "NK cell∗" OR "T helper cell∗" OR "th cell∗" OR "T cell∗" OR "Treg∗" OR CTL OR macrophage∗ OR leukocyt∗ OR "B cell∗" OR "myeloid∗" OR inflammat∗ OR Immuno∗ OR Flow cyto∗" OR "Plasma cell∗" OR "Monocyte∗" OR Fibroblast OR Interleukin∗ OR "Antigen presenting"):ti,ab,kw	13,371,232
#3	(('squamous cell carcinoma'/exp OR ("squamous cell carcinoma∗" OR "squamous carcinoma∗" OR "Epidermoid Carcinoma∗" OR "Planocellular Carcinoma∗" OR "Plano cellular Carcinoma∗" OR Squamous∗):ti,ab,kw) AND (cervical∗ OR cervix∗ OR Endocervical):ti,ab,kw)	36,667
#2	(('adenocarcinoma'/exp OR "adenocarcinoma":ti,ab,kw) AND (cervical∗ OR cervix∗ OR Endocervical):ti,ab,kw)	19,745
#1	'uterine cervix tumour'/exp OR ("cervical cancer∗" OR "cervical tumo∗" OR "cervical malignanc∗" OR "cervical neoplasm∗" OR "cervical carcinoma∗" OR "cervical adenocarcinoma" OR "cervical intraepithelial neoplasia" OR "cervical metasta∗" OR "cervix cancer∗" OR "cervix tumo∗" OR "cervix malignanc∗" OR "cervix neoplasm∗" OR "cervix carcinoma∗" OR "cervix adenocarcinoma" OR "cervix squamous cell carcinoma∗" OR "cervix intraepithelial neoplasia" OR "cervical squamous cell carcinoma∗"):ti,ab,kw	175,778

**Table A.3:** Search Strategy Web of Science.

Search	Query	Results
#5	Search: #1 AND #2 AND #3 AND #4	900
#4	TS=(Immun∗ OR "programmed death ligand∗" OR "programmed cell death ligand" OR "programmed death 1∗" OR "programmed cell death 1∗" OR Lymphoid∗ OR TLS OR Basophil∗ OR Neutrophil∗ OR "Interferon gamma" OR Cytokine∗ OR Chemokine∗ OR CAF OR Eosinophil∗ OR "Toll-like receptor∗" OR IHC OR Immunohistochem∗ OR FFPE OR "Formalin-fixed paraffin embedded" OR TIL OR lymphocyt∗ OR "tumour infiltrat∗" OR "tumour infiltrat∗" OR "PD 1″ OR "PD L1" OR PD1 OR PDL1 OR "T cell∗" OR "CTLA∗" OR CTLA4∗ OR "dendritic cell∗" OR CD4 OR CD8 OR "CD 4∗" OR "CD 8∗" OR "natural killer cell∗" OR "NK cell∗" OR "T helper cell∗" OR "th cell∗" OR "T cell∗" OR "Treg∗" OR CTL OR macrophage∗ OR leukocyt∗ OR "B cell∗" OR "myeloid∗" OR inflammat∗ OR Immuno∗ OR "Flow cyto∗" OR "Plasma cell∗" OR "Monocyte∗" OR Fibroblast OR Interleukin∗ OR "Antigen presenting")	6,039,181
#3	TS=((("squamous cell carcinoma∗" OR "squamous carcinoma∗" OR "Epidermoid Carcinoma∗" OR "Planocellular Carcinoma∗" OR "Plano cellular Carcinoma∗" OR Squamous∗)) AND (cervical∗ OR cervix∗ OR Endocervical))	28,903
#2	TS=(("adenocarcinoma") AND (cervical∗ OR cervix∗ OR Endocervical))	10,969
#1	TS=("cervical cancer∗" OR "cervical tumo∗" OR "cervical malignanc∗" OR "cervical neoplasm∗" OR "cervical carcinoma∗" OR "cervical adenocarcinoma" OR "cervical intraepithelial neoplasia" OR "cervical metasta∗" OR "cervix cancer∗" OR "cervix tumo∗" OR "cervix malignanc∗" OR "cervix neoplasm∗" OR "cervix carcinoma∗" OR "cervix adenocarcinoma" OR "cervix squamous cell carcinoma∗" OR "cervix intraepithelial neoplasia" OR "cervical squamous cell carcinoma∗")	108,719

**Table A.4:** Search Strategy Cochrane Library.

Search	Query	Results
#5	#1 AND #2 AND #3 AND #4	187
#4	(Immun∗ OR programmed death ligand∗ OR programmed cell death ligand OR programmed death 1∗ OR programmed cell death 1∗ OR Lymphoid∗ OR TLS OR Basophil∗ OR Neutrophil∗ OR Interferon gamma OR Cytokine∗ OR Chemokine∗ OR CAF OR Eosinophil∗ OR Toll-like receptor∗ OR IHC OR Immunohistochem∗ OR FFPE OR Formalin-fixed paraffin embedded OR TIL OR lymphocyt∗ OR tumour infiltrat∗ OR tumour infiltrat∗ OR PD 1 OR PD L1 OR PD1 OR PDL1 OR T cell∗ OR CTLA∗ OR CTLA4∗ OR dendritic cell∗ OR CD4 OR CD8 OR CD 4∗ OR CD 8∗ OR natural killer cell∗ OR NK cell∗ OR T helper cell∗ OR th cell∗ OR T cell∗ OR Treg∗ OR CTL OR macrophage∗ OR leukocyt∗ OR B cell∗ OR myeloid∗ OR inflammat∗ OR Immuno∗ OR Flow cyto∗ OR Plasma cell∗ OR Monocyte∗ OR Fibroblast OR Interleukin∗ OR Antigen presenting):ti,ab,kw	347275
#3	(((squamous cell carcinoma∗ OR squamous carcinoma∗ OR Epidermoid Carcinoma∗ OR Planocellular Carcinoma∗ OR Plano cellular Carcinoma∗ OR Squamous∗)) AND (cervical∗ OR cervix∗ OR Endocervical)):ti,ab,kw	1701
#2	((adenocarcinoma) AND (cervical∗ OR cervix∗ OR Endocervical)):ti,ab,kw	675
#1	(cervical cancer∗ OR cervical tumo∗ OR cervical malignanc∗ OR cervical neoplasm∗ OR cervical carcinoma∗ OR cervical adenocarcinoma OR cervical intraepithelial neoplasia OR cervical metasta∗ OR cervix cancer∗ OR cervix tumo∗ OR cervix malignanc∗ OR cervix neoplasm∗ OR cervix carcinoma∗ OR cervix adenocarcinoma OR cervix squamous cell carcinoma∗ OR cervix intraepithelial neoplasia OR cervical squamous cell carcinoma∗):ti,ab,kw	9278

**Table B.1:** Study characteristics.

Author	Year	Country	Study design	Number of SCC	Number of AC	Number of patients	Stage of disease	Type of material	Techniques used	Immune markers
Baek MH et al. [22]	2024	Korea	Cohort	203	64	267	Advanced, recurrent, metastatic	Tumour FFPE	IHC	PD-L1
Chiba Y et al. [23]	2024	Japan	Cohort	67	39	106	Early, Locally Advanced, Advanced	Tumour FFPE	IHC	PD-L1
Dai Y et al. [24]	2017	China	Cohort	33	15	48	Early, Locally Advanced	Tumour FFPE	IHC	CXCR4, CCR7
De Jaeghere EA et al. [25]	2023	Belgium	Cohort	12	5	17	Early, Locally Advanced, Advanced	Tumour FFPE	IHC	PD-L1, CD3
Enwere EK et al. [26]	2017	Canada	Cohort	106	14	120	Early, Locally Advanced, Advanced	Tumour FFPE	IHC	PD-L1
Ferns DM et al. [27]	2016	Netherlands	Cohort	103	33	136	Early, Locally Advanced	Tumour FFPE	IHC	HLA-A/B/C/E/G
Fujiwaki R et al. [28]	2003	Japan	Cohort	38	9	47	Early, Locally Advanced, Advanced	Tumour FFPE, Blood	ELISA, WB, IHC	IL-1ra
Guo S et al. [29]	2024	China	Cross-Sectional	98	12	110	Early, Locally Advanced	Tumour FFPE	IHC	CD8
Heeren AM et al. [15]	2016	Netherlands	Cohort	252	80	332	Early	Tumour FFPE	IHC	PD-L1, CD163, TAMs
Hosseini MS et al. [30]	2024	Iran	Cross-Sectional	48	16	64	Early	Tumour FFPE	IHC	PD-L1
Huang RSP et al. [31]	2021	U.S.	Cohort	437	58	495	NR	Tumour FFPE	IHC	PD-L1
Jordanova ES et al. [32]	2008	Netherlands	Cohort	89	26	115	Early	Tumour FFPE	IHC	CD3, CD4, CD8, Tregs
Karpathiou G et al. [33]	2020	France	Cross-Sectional	19	27	46	Early, Locally Advanced, Advanced	Tumour FFPE	IHC	PD-L1, CTLA-4
Kim M et al. [68]	2017	Korea	Cohort	18	9	27	Early	Tumour FFPE	IHC	PD-L1
Lathika AS et al. [35]	2021	India	Cohort	81	16	97	Early, Locally Advanced, Advanced	Tumour FFPE	IHC	PD-L1
Li C et al. [36]	2023	China	Cross-Sectional	3	3	6	NR	Fresh tumour	scRNAseq	CD4, CD8, naive CD4, naive CD8, CD8 EM, CD8 CM, NK-cells, TAMs (M1/M2-like), CD163, PD-L1, CTLA-4, ICOS, CD28, CXCL9/10/11, CCL13/18, CXCL3/CXCL5, IFN-γ, TGFβ1
Li J et al. [37]	2023	China	Cohort	254	82	336	Early, Locally Advanced, Advanced	Tumour FFPE, Blood	IHC	PD-L1
Li L et al. [38]	2021	China	Cross-Sectional	58	12	70	Early	Tumour FFPE	IHC	VISTA, FOXP3
Li Y et al. [39]	2017	China	Cohort	74	16	90	Early, Locally Advanced	Tumour FFPE, Blood, Cell Lines	IHC, ELISA, MTT, migration	PD-L1, B7-H3
Lim MC et al. [40]	2024	Korea	Clinical Trial	50	15	65	Recurrent, advanced	Tumour FFPE	IHC	PD-L1
Lin S et al. [41]	2023	China	Cohort	5	3	8	Early, Locally Advanced	Fresh tumour, tumour FFPE	snRNAseq, mIF	CD3, CD4, CD8, Tregs, (memory) B cells, plasma cells, myeloid cells, macrophages
Liu B et al. [42]	2024	China	Cohort	44	19	63	Early, Locally Advanced	Tumour FFPE, TMA	RNAseq, IHC, mIF	TILs, CD3, CD4, CD8, naive CD4, memory CD4, Tregs, CD8, naive CD8, CD8 EM, NK cells, B cells, myeloid cells, macrophages, DCs, pDCs, PD-L1, CTLA-4, PD-1, PD-L2, LAG-3, TIGIT, IL-10, TGFβ, HLA class I/II
Liu J et al. [43]	2022	China	Cohort	67	26	93	Early, Locally Advanced, Advanced	Tumour FFPE	IHC	PD-L1
Mancusi de Carvalho JP et al. [44]	2020	Brazil	Cohort	45	26	71	Early	Tumour FFPE	IHC	TILs
Nakano T et al. [45]	1992	Japan	Cohort	216	59	275	SCC Locally Early, Locally Advanced, Advanced	Tumour FFPE	IHC	TILs, CD3, Langerhans cells
O'Malley DM et al. [46]	2021	U.S.	Clinical Trial	85	48	133	Metastatic, persistent, recurrent	Tumour FFPE	IHC	PD-L1
Omenai SA et al. [47]	2022	Nigeria	Cross-Sectional	155	12	167	NR	Tumour FFPE	IHC	PD-L1
Qiu J et al. [48]	2023	China	Cohort	13	10	23	Early	Fresh tumour, Tumour FFPE	scRNAseq, IHC	CD4, CD8, TIGIT, HLA-E
Reddy OL et al. [49]	2017	U.S.	Cross-Sectional	74	12	86	NR	Tumour FFPE	IHC	PD-L1
Rischin D et al. [50]	2020	Australia	Cohort	112	43	155	NR	Tumour FFPE	IHC, TCGA mRNA expression	PD-L1
Rotman J et al. [51]	2020	Netherlands	Cohort	44	14	58	Early	Fresh tumour, Tumour FFPE	IHC, mRNA expression	PD-L1, PD-L2, CD80, macrophages
Rotman J et al. [17]	2020	Netherlands	Cohort	20	16	36	Early, Locally Advanced	Fresh tumour, TDLN	Flow cytometry, mRNAseq, CBA assay	CD45, CD3, TILs, CD4, CD8, EpCAM, Tregs, naïve CD8, CD8 CM, CD8 EM, DCs, cDC1, PD-1, CTLA-4, LAG-3, TIM-3, CXCL8/9/10/11, CCL4
Salarzaei M et al. [52]	2024	Netherlands	Cohort	11	10	21	Early	Tumour FFPE	Gene expression analysis	CD45, CD3, CD4, CD8, NK-cells, B-cells, macrophages, DCs, PD-L1, CTLA-4
Samuels S et al. [53]	2016	Netherlands	Cohort	60	64	124	Early	Tumour FFPE	IHC	HLA-DR
Santin AD et al. [54]	1997	U.S.	Cross-Sectional	4	4	8	NR	Fresh tumour	ELISA	TGF-B
Sayhan S et al. [55]	2021	Türkiye	Cross-Sectional	27	6	33	NR	Tumour FFPE	IHC	PD-L1, FoxP3
Sheu BC et al. [56]	1999	Taiwan	Cohort	20	10	30	Early	Blood, Fresh tumour	Flow cytometry	CD45, CD3, CD4, CD8, B-cells (CD19), NK-cells (CD56)
Takano H et al. [57]	1996	Japan	Cross-Sectional	66	4	70	NR	Blood, Cell lines	ELISA	IL-6
Tewari KS et al. [58]	2022	U.S.	RCT	208	46	254	Recurrent or metastatic	Tumour FFPE	IHC	PD-L1
Wild CM et al. [59]	2023	Germany	Cohort	191	47	238	Early, Locally Advanced, Advanced	Tumour FFPE	IHC	TILs
Zhang Q et al. [60]	2021	China	Cohort	406	146	552	Early	Tumour FFPE	IHC	B7-H3, PD-L1
Zhang Y et al. [61]	2017	China	Cohort	58	8	66	Early	Blood	Flow cytometry, ELISA	PD-L1, CD14, monocytes
Zong L et al. [62]	2022	China	Cohort	491	115	606	Early	Tumour FFPE	IHC	PD-L1, B7-H3, B7-H4, VISTA

CBA, Cytometric Beat Assay; DCs, dendritic cells; ELISA, Enzyme-Linked Immunosorbent Assay; FFPE, Formalin-Fixed Paraffin Embedded; IHC, Immunohistochemistry; mIF, Multicolor Immunofluorescence; MTT, 3-(4,5-Dimethylthiazol-2-yl)-2,5-Diphenyltetrazolium Bromide; NR, Not Reported; scRNAseq, Single Cell RNA Sequencing; TCGA, The Cancer Genome Atlas; TDLN, tumour-draining lymph node; TMA, Tissue Microarray; Tregs, regulatory T cells; U.S., United States of America; WB, Western Blot.

**Table C.1:** Critical Appraisal.

Author	Year	1.	2.	3.	4.1	4.2	5.	Quality
Baek MH et al. [22]	2024	+	+	+	+	+	+	High
Chiba Y et al. [23]	2024	+	+	+	+	+/−	–	Moderate
Dai Y et al. [24]	2017	+	+	+	+	+	+	High
De Jaeghere EA et al. [25]	2023	+	–	+/−	+	+	+	Moderate
Enwere EK et al. [26]	2017	+	+/−	+	+	+	+	High
Ferns DM et al. [27]	2016	+	+	+	+	+	+	High
Fujiwaki R et al. [28]	2003	+/−	+	+	+	+	+	High
Guo S et al. [29]	2024	+/−	+	+	+	+	+	High
Heeren AM et al. [15]	2016	+	+	+	+	+	+	High
Hosseini MS et al. [30]	2024	+	+	+	+	+	+	High
Huang RSP et al. [31]	2021	+	+	+/−	+	+	+	High
Jordanova ES et al. [32]	2008	+	+	+	+	+	+	High
Karpathiou G et al. [33]	2020	+	+	+	+	+	+	High
Kim M et al. [68]	2017	+	+	+	+	+	+	High
Lathika AS et al. [35]	2021	+	+	+	+	+	+	High
Li C et al. [36]	2023	+	+	+	+	+	+	High
Li J et al. [37]	2023	+	+	+	+	+	+	High
Li L et al. [38]	2021	+	+	+	+	+	+	High
Li Y et al. [39]	2017	+/−	+	–	+	+	+	Moderate
Lim MC et al. [40]	2024	+	+	+	+	+	+	High
Lin S et al. [41]	2023	+/−	+	+	+	+	+	High
Liu B et al. [42]	2024	+/−	–	+	+	+	+	Moderate
Liu J et al. [43]	2022	+/−	+	–	+	+	+	Moderate
Mancusi de Carvalho JP et al. [44]	2020	+	+	+	+	+	+	High
Nakano T et al. [45]	1992	–	+	+	+	+	–	Low
O'Malley DM et al. [46]	2021	+	+	+	+	+	+	High
Omenai SA et al. [47]	2022	+	+	+	+	+	+	High
Qiu J et al. [48]	2023	+	+	+	+	+	+	High
Reddy OL et al. [49]	2017	–	–	+	+	+	+	Low
Rischin D et al. [50]	2020	–	–	+	+	+	+	Low
Rotman J et al. [51]	2020	+	+	+	+	+	+	High
Rotman J et al. [17]	2020	+	+	+	+	+	+	High
Salarzaei M et al. [52]	2024	+	+	+	–	–	+	Low
Samuels S et al. [53]	2016	–	–	+	+	+	+	Low
Santin AD et al. [54]	1997	+/−	–	+	+/−	+	+	Low
Sayhan S et al. [55]	2021	+/−	–	+	+/−	+	–	Low
Sheu BC et al. [56]	1999	+/−	+/−	+	+/−	+	+	Moderate
Takano H et al. [57]	1996	–	–	+/−	+/−	+	+/−	Low
Tewari KS et al. [58]	2022	+	+/−	+	+	+	+	High
Wild CM et al. [59]	2023	+	+	+	+	+	+	High
Zhang Q et al. [60]	2021	+	+	+	+	+	+	High
Zhang Y et al. [61]	2017	+	+	+	+	+	+	High
Zong L et al. [62]	2022	+	–	+	+	+	+	Moderate

Score: '+' = 1; '+/−' = 0.5; '-' = 0. High = 5.5–6, Moderate = 4.5–5.5, Low = < 4.5.

## Data Availability

Data will be made available on request.
